# Improving children’s health and development in British Columbia through nurse home visiting: a randomized controlled trial protocol

**DOI:** 10.1186/s12913-016-1594-0

**Published:** 2016-08-04

**Authors:** Nicole L. A. Catherine, Andrea Gonzalez, Michael Boyle, Debbie Sheehan, Susan M. Jack, Kaitlyn A. Hougham, Lawrence McCandless, Harriet L. MacMillan, Charlotte Waddell, For the British Columbia Healthy Connections Project Scientific Team

**Affiliations:** 1 Children’s Health Policy Centre, Faculty of Health Sciences, Simon Fraser University, Room 2431, 515 West Hastings Street, Vancouver, BC V6B 5 K3 Canada; 2 Offord Centre for Child Studies, Faculty of Health Sciences, McMaster University, Hamilton, ON Canada; 3 Department of Psychiatry and Behavioral Neurosciences, Faculty of Health Sciences, McMaster University, Hamilton, ON Canada; 4 School of Nursing, Faculty of Health Sciences, McMaster University, Hamilton, ON Canada; 5 Faculty of Health Sciences and Department of Statistics and Actuarial Science, Faculty of Science, Simon Fraser University, Vancouver, BC Canada

**Keywords:** Nurse-family partnership, Randomized controlled trial, Early child development, Prevention, Child injuries

## Abstract

**Background:**

Nurse-Family Partnership is a nurse home visitation program that aims to improve the lives of young mothers and their children. The program focuses on women who are parenting for the first time and experiencing socioeconomic disadvantage. Nurse visits start as early in pregnancy as possible and continue until the child reaches age two years. The program has proven effective in the United States – improving children’s mental health and development and maternal wellbeing, and showing long-term cost-effectiveness. But it is not known whether the same benefits will be obtained in Canada, where public services differ. The British Columbia Healthy Connections Project therefore involves a randomized controlled trial evaluating Nurse-Family Partnership’s effectiveness compared with existing (usual) services in improving children’s mental health and early development and mother’s life circumstances. The trial’s main aims are to: reduce childhood injuries by age two years (primary outcome indicator); reduce prenatal nicotine and alcohol use; improve child cognitive and language development and behaviour at age two years; and reduce subsequent pregnancies by 24 months postpartum. Potential explanatory factors such as maternal mental health (including self-efficacy) are also being assessed, as is the program’s impact on exposure to intimate-partner violence. To inform future economic evaluation, data are also being collected on health and social service access and use.

**Methods/design:**

Eligible and consenting participants (*N* = 1040) are being recruited prior to 28 weeks gestation then individually randomized to receive existing services (comparison group) or Nurse-Family Partnership plus existing services (intervention group). Nurse-Family Partnership is being delivered following fidelity guidelines. Data are being collected during in person and telephone interviews at: baseline; 34–36 weeks gestation; and two, 10, 18 and 24 months postpartum. Additional data will be obtained via linkages from provincial datasets. Recruitment commenced in October 2013 and will continue for approximately three years.

**Discussion:**

This trial will provide important information about the generalizability of Nurse-Family Partnership to the Canadian context. Findings will be published in peer-reviewed journals and shared with policymakers and practitioners through extensive public health collaborations already underway.

**Trial registration:**

Registered July 18, 2013 with ClinicalTrials.gov Identifier: NCT01672060.

## Background

### Nurse-family partnership

Developed nearly 40 years ago by Olds and colleagues in the United States (US), Nurse-Family Partnership (NFP) is a nurse home visitation program that aims to improve the lives of young, first-time mothers and their children who are experiencing socioeconomic disadvantage [1–4]. NFP is based on theories of human ecology, self-efficacy and attachment and aims to improve prenatal health and parenting while also improving life course outcomes for both children and mothers. The program concentrates on first-time mothers who are theorized as being more receptive to parenting education and support [2]. NFP starts as early in pregnancy as possible (with first visits no later than the end of the 28th week of gestation) and continues until children reach age two years [1–4].

In the NFP program, nurses provide frequent home visits. The goals for these visits include: 1) improving prenatal health behaviours; 2) increasing sensitive and competent parenting; 3) reducing the risks of child behaviour and other mental health problems; and 4) helping mothers improve their economic self-sufficiency, e.g., through fewer subsequent pregnancies, longer intervals between pregnancies and greater workforce participation. Nurses receive extensive education and resources to use in the home visits, as well as ongoing supervision to ensure that they are not only highly skilled, but also well supported [1–4]. Nurses have proven to be the most successful home visitors because of their training and expertise, as well as their professional credibility with families [5].

NFP has been evaluated in three randomized controlled trials (RCTs) in the United States (US) – in Elmira, New York, Memphis, Tennessee and Denver, Colorado. Results from these RCTs have been reported in numerous articles, as have long-term follow-up findings over 20 years [5–20]. NFP has shown several robust and enduring effects on maternal and child health outcomes, including decreased mortality due to preventable causes [5–20]. Two independent research groups have also conducted comprehensive cost-benefit analyses of NFP in the US. The Rand Corporation estimated net returns of US $2.88 for every dollar invested, with returns for the highest-risk families nearly doubled at US $5.70 for every dollar invested [21]. Similarly, the Washington State Institute for Public Policy estimated a return on investment of more than US $17,000 for every family served [22]. Both evaluations factored in averted costs across multiple public sectors over 10–15 years, including reduced healthcare, income assistance and child protection spending. Notably, averted public expenditures were greatest for the most disadvantaged mothers and children, underscoring the importance of offering NFP to those at higher risk [23].

Results are also now available from trials of NFP conducted outside the US [24, 25]. Findings from a trial conducted in the Netherlands indicated that in comparison to existing health and social services, NFP reduced prenatal smoking, increased breastfeeding, reduced child protection reports, and reduced exposure to intimate-partner violence [26–28]. However, an RCT evaluating NFP in England demonstrated no additional benefits for children or mothers compared to usual health and social services [29]. The differing findings across the American, Dutch and English trials underscore the need to conduct RCTs in countries outside the US prior to widespread implementation – to ascertain NFP’s effectiveness in comparison with existing services for this disadvantaged population [30]. Interventions may well have different outcomes in different contexts, particularly if existing services differ [31, 32].

### Evaluating nurse-family partnership in Canada

NFP has been shown to decrease childhood injuries and improve children’s mental health and development [5–19]. In Canada, an estimated 13 % of children (or nearly 700,000) are affected by mental disorders at any given time, with these disorders constituting a leading health problem for children [33]. Mental disorders in childhood then generally persist into adulthood, with a wide range of negative consequences [34–36]. Anxiety, substance use, conduct and depressive disorders are among the most common childhood disorders – that may be preventable through programs such as NFP [33]. Younger mothers are also more likely to experience limited education and low income – factors associated with increased risk of child injuries and poor child cognitive and behavioural development [35, 37–40]. Therefore it is vital to intervene as early as possible to reduce avoidable adversities and to avert long-term consequences such as preventable mental disorders. Yet NFP has never been tested in Canada.

Work began in Canada in 2008 with an Ontario-based pilot study, in keeping with the international replication guidelines for NFP [30, 41]. This pilot involved first adapting NFP nurse and participant educational materials to the Canadian context. Then NFP’s feasibility and acceptability were assessed with over 100 mothers and children. NFP was well received by these mothers – and by nurses, family members and community partners, laying the foundation for conducting an RCT in British Columbia (BC) [41, 42].

In BC, children’s mental health has long been a priority, starting in 2003 with a five-year children’s mental health plan, and continuing in 2010 with a 10-year mental health plan for the entire provincial population [43, 44]. The 10-year plan made prevention a high priority, featuring nurse home visitation for disadvantaged first-time mothers and their children as a central initiative. Planning for the BC Healthy Connections Project or BCHCP – involving an RCT evaluating NFP – commenced in 2010. The RCT is being jointly led by researchers at Simon Fraser University (SFU) and McMaster University – in close collaboration with senior policymakers in the BC Ministries of Health and Children and Family Development, and with four regional BC Health Authorities (Fraser Health, Interior Health, Island Health and Vancouver Coastal Health). (A fifth regional Health Authority, Northern Health, also participates in the collaboration, but not in the RCT.)

The RCT is accompanied by two adjunctive studies – a Process Evaluation investigating NFP’s nursing implementation in BC, and the Healthy Foundations Study, a biological evaluation of NFP’s impact on child health [42, 45]. Five Health Authorities are participating in the Process Evaluation, while Fraser Health and Vancouver Coastal Health are participating in the Healthy Foundations Study.

## Methods/design

### Research aim

The aim of the trial is to evaluate NFP’s effectiveness compared with existing services in improving children’s mental health and early development and mother’s life circumstances. One primary outcome indicator has been identified: the average number of physician encounters per child for (intentional and unintentional) injuries, measured in community/outpatient, emergency and hospital settings from birth through to age two years. Four secondary outcome indicators have also been identified: prenatal nicotine and alcohol use; child cognitive development (language and cognitive ability) at age two years; child behaviour (internalizing and externalizing problems) at age two years; and the incidence of subsequent pregnancies by 24 months postpartum. (See Tables 4 and 5.) Potential explanatory factors such as maternal mental health (including self-efficacy) are also being assessed, as is the program’s impact on exposure to intimate-partner violence (IPV), a novel addition through the Canadian NFP curriculum [46]. Data are also being collected on health and social service access and use – to set the stage for long-term economic evaluation.

### Study design

Prior to 28 weeks gestation, eligible and consenting participants (N = 1040) complete a baseline assessment and are individually randomized to receive existing services (comparison group) or NFP plus existing services (intervention group). NFP is being delivered following fidelity guidelines – with women being offered the program starting as early as possible in pregnancy and continuing until children reach age two years [41, 47]. The trial opened to enrollment in October 2013 and enrollment will continue for approximately three years, with data collection ongoing for 2.5 years thereafter or until the last enrolled participant’s child reaches age two years.

### Settings

BC has a population of 4.6 million dispersed across an area of nearly 950,000 km^2^ [48]. Regional Health Authorities are responsible for providing all local public health and healthcare services, with the Ministry of Health overseeing province-wide standards and providing funding [49]. Regarding specific settings for the RCT, each regional Health Authority is further divided into Local Health Areas that delineate smaller geographic foci for service delivery. The BC Ministry of Health and the Health Authorities together identified particular Local Health Areas where NFP delivery would be feasible, i.e., where there was sufficient population to justify the public health nursing resources needed to deliver NFP. As part of the BCHCP’s policy and practice collaborations, Health Authorities are hiring and overseeing the public health nurses (PHNs), who then receive their NFP education, preparing them to deliver the program to the intervention group as part of the RCT. The four participating Health Authorities are also the RCT’s main referral source. Table 1 describes the participating Local Health Areas that include a range of urban, suburban and smaller communities.

**Table 1:** Participating local health areas

Health authority	Local health area
Fraser Health	Abbotsford, Burnaby, Chilliwack, Coquitlam, Delta, Langley, Maple Ridge, Mission, New Westminster, South Surrey/White Rock, Surrey
Interior Health	Central Okanagan, Kamloops, Vernon
Island Health	Cowichan/Lake Cowichan, Greater Victoria, Nanaimo/Ladysmith, Saanich, Sooke
Vancouver Coastal Health	Downtown Eastside, North East, South, North Vancouver/West Vancouver-Bowen Island, Richmond, Vancouver City Centre, Westside/Midtown

### Public health nurse preparation

Nurse preparation is critical, as these practitioners are central to the NFP intervention. Starting in 2012, Health Authorities recruited a cadre of approximately 75 experienced PHNs then sponsored intensive education to prepare them for delivering NFP through the RCT, and to prepare their supervisors. (Note that BC PHNs typically hold baccalaureate degrees, in addition to having a decade or more of public health experience.) The US NFP National Service Office provided BC’s NFP education initially, but BC is now developing the capacity to provide this education locally. A novel aspect of this effectiveness trial is the evaluation of NFP’s impact on maternal exposure to IPV. Previous NFP trials have indicated that when nurse-visited women reported high rates of IPV, reductions in child maltreatment significantly declined [16]. Therefore, IPV modules were included in the BC nurses’ education [46].

NFP PHNs and supervisors also participated in an education pilot to consolidate and hone their skills by delivering the full NFP program to a small caseload of young women – who served as “guiding clients.” Identified through usual public health referral sources, nearly 300 pregnant women provided written informed consent to participate as guiding clients. They have received NFP, but no research data were collected. All subsequently recruited PHNs no longer have guiding clients, but rather directly observe and practice through a mentorship model now led by experienced BC NFP nurses.

### Nurse-family partnership intervention

PHNs deliver NFP to eligible and consenting women through regular home visits throughout the pregnancy and continuing until the child’s second birthday – up to 64 visits in total over 2.5 years (if the program starts by the 16th week of pregnancy). Each visit typically lasts 60–90 min. PHNs use visit-to-visit guidelines that were adapted for Canadian settings [41, 47]. Beyond their intensive NFP education, PHNs also receive regular individual and team reflective supervision and support to ensure fidelity to essential elements of the NFP program model. Table 2 outlines the Canadian NFP model elements [41, 47]. As part of these elements, each participant in the NFP intervention arm is also assigned to one specific PHN for the duration of program delivery (wherever possible) to facilitate a close and continuing relationship [50]. For the duration of RCT recruitment, NFP is only available in BC through the trial.

**Table 2:** Canadian nurse-family partnership model elements

Client characteristics	
1.	Client participates voluntarily in the Nurse-Family Partnership program.
2.	Client is a first-time mother.
3.	Client meets socioeconomic disadvantage criteria at intake.
4.	Client is enrolled in the program early in her pregnancy and receives her first home visit no later than the end of the 28th week of pregnancy.
Intervention context	
5.	Client is visited one-to-one: one public health nurse to one first-time mother/family.
6.	Client is visited in her home.
7.	Client is visited throughout her pregnancy and the first two years of her child’s life in accordance with the current Nurse-Family Partnership guidelines.
Expectations of nurses and supervisors	
8.	Public health nurses and nurse supervisors are registered professional nurses with a minimum of a baccalaureate degree in nursing.
9.	Public health nurses and nurse supervisors complete core educational sessions required by the University of Colorado and deliver the intervention with fidelity to the NFP model.
Application of the intervention	
10.	Public health nurses, using professional knowledge, judgment and skill, apply the Nurse-Family Partnership Visit-to-Visit Guidelines, individualizing them to the strengths and challenges of each family and apportioning time across defined program domains.
11.	Public health nurses apply the theoretical framework that underpins the program, emphasizing Self-Efficacy, Human Ecology and Attachment theories, through current clinical methods.
12.	A full-time public health nurse carries a caseload of no more than 20 active clients.
Reflection and clinical supervision	
13.	A full-time nurse supervisor provides supervision to no more than eight individual public health nurses.
14.	Nurse supervisors provide public health nurses clinical supervision with reflection, demonstrate integration of the theories, and facilitate professional development essential to the public health nurse role through specific supervisory activities including one-to-one clinical supervision, case conferences, team meetings, and field supervision.
Program monitoring and use of data	
15.	Public health nurses and nurse supervisors collect data as specified by the University of Colorado (or provincial equivalents) and use Nurse-Family Partnership reports to guide their practice, assess and guide program implementation, inform clinical supervision, enhance program quality, and demonstrate program fidelity.
Sponsoring agency	
16.	An Nurse-Family Partnership Implementing Agency is located in and operated by an organization known in the community for being a successful provider of prevention services to low-income families.
17.	An Nurse-Family Partnership Implementing Agency convenes a long-term Community Advisory Board that meets at least quarterly to establish a community support system for the program and to promote program quality and sustainability.
18	Adequate support and structure shall be in place to support public health nurses and nurse supervisors to implement the program and to assure that data are accurately entered into the database in a timely manner.

### Existing services

Within BC’s universal healthcare system, all trial participants, including those receiving NFP, are eligible to receive existing services (usual care) provided in their Health Authority. Existing health services for pregnant women and young children vary across BC but may include: primary healthcare services, provided by physicians and also by midwives in some circumstances; specialist physician services; public health programs including pregnancy screening and outreach, prenatal classes and brief forms of home visiting by (non-NFP) nurses or paraprofessionals; and a variety of targeted and universal parenting and early child development programs. Health Authorities also provide adult mental healthcare including substance misuse and harm reduction services. In BC, all basic public health services are typically provided at no cost. There is also no cost for BC’s universal healthcare services including physician, emergency and hospital visits – although there may be costs associated with prescription medications, unless the family meets low-income eligibility criteria [49]. A wide array of related social services are also offered in BC by the federal and provincial governments, municipalities and local charitable groups including: employment assistance; child benefits; income assistance; education assistance; crisis interventions; child protection programs; justice services; shelter and housing supports; and food banks. All these health and social services are currently offered in BC. However, it is not currently known how much local availability varies and whether young mothers and their children actually access and use these services – particularly those experiencing socioeconomic disadvantage. Consequently, data on health and social service access and use are being gathered for all participants throughout the trial.

### Eligibility criteria

For this RCT, eligibility criteria were informed by: 1) NFP model elements; [41, 47] 2) criteria used in previous NFP RCTs; [8, 11, 14, 24, 25] and 3) research literature indicating that maternal socioeconomic disadvantage is associated with child injuries [38, 39, 51]. Participant inclusion and exclusion criteria are outlined in Table 3.

**Table 3:** Participant inclusion/exclusion criteria

Women are eligible to participate if they meet *all* inclusion criteria at time of baseline interviews	
1.	Age 24 years or younger
2.	First birth. Women are eligible if a previous pregnancy ended in termination, miscarriage or stillbirth, or if previous parenting involved step-parenting only
3.	Less than 28 weeks gestation. Women are recruited prior to 28 weeks gestation to ensure that participants randomized to NFP receive their first home visit by the end of the 28th week of gestation, according to NFP fidelity requirements.
4.	Competent to provide informed consent, including conversational competence in English
5.	Experiencing socioeconomic disadvantage
•	Age 19 or younger
•	Age 20–24: Meets 2 of 3 indicators: *Lone parent*; *less than grade 12*; or *low income* which requires one or more of:
	i. Receiving Medical Services Plan Premium Assistance, disability assistance or other income assistance;
	ii. Finding it very difficult to live on total household income with respect to food or rent; [60] or
	iii. Homeless, defined as living on the streets, living in a place not meant as a long-term dwelling (e.g., car or tent), staying in a shelter, or staying somewhere temporarily with no permanent address (e.g., “couch surfing”) [61, 62].
Women are ineligible to participate if they meet *any* exclusion criteria at time of baseline interviews	
1.	Planning to have the child adopted
2.	Planning to leave the BCHCP catchment area (designated Local Health Areas) for three months or longer during the trial.

### First nations and aboriginal women and children

In BC, First Nations and Aboriginal or Indigenous peoples, including Métis peoples, may live on designated “reserve” lands or in communities outside these lands. A pan-BC First Nations Health Authority holds responsibility for all public health and healthcare programs regardless of location – with supports from the BC Government and the regional Health Authorities [48]. Respecting this governance process, for this RCT, all eligible women self-identifying as First Nations or Aboriginal or Indigenous who are living “off reserve” at time of enrollment (i.e., randomization into the trial) are invited to participate if they choose. However, First Nations and Aboriginal or Indigenous women who are living “on reserve” at the time of enrollment are not eligible for the RCT.

### Recruitment

Across BC, Health Authorities have established prenatal registries to promote referrals to public health. The main referral sources to these registries are primary healthcare providers including physicians and midwives, as well as youth-serving agencies including schools. The goal is to ensure that all pregnant women are referred to Health Authority public health teams as early as possible in gestation. Once women are referred to prenatal registries, PHNs first offer existing services and only after this, screen and refer potentially eligible women to the Scientific Team at SFU.

Scientific field interviewers (who are involved in recruitment and data collection, but masked to intervention assignment), then contact women by telephone to introduce the RCT, assess eligibility and schedule an in-person interview. During the interview, the scientific field interviewers confirm eligibility, obtain written informed consent and administer the baseline assessment. Next, a senior member of the Scientific Team (who is not involved in recruitment or data collection) follows a strict randomization protocol outlined below to assign participants to either the intervention or comparison groups and informs women and referring Health Authorities of the randomization allocation. Health Authorities then initiate provision of NFP (and existing services) accordingly.

### Randomization

An unpredictable randomized sequence protocol was designed to allocate women in a 1:1 ratio to either the comparison (existing services) or intervention (NFP plus existing services) groups, and to ensure adequate concealment of randomization assignment [52]. A separate randomization schedule was generated for each of the 26 participating Local Health Areas within the Health Authorities using constrained randomization (permuted block design), wherein the smaller of the two block sizes is applied in areas where fewer than 18 women a year are expected to be randomized. The specific block sizes were chosen as the best trade-off between the loss of power associated with the use of block randomization and ensuring a balance in the number of women allocated to each trial group in the smaller sites, while also enabling management of NFP nursing workloads in both small and large Local Health Areas. The Scientific Team follows strict protocols for safeguarding administration of the group assignment schedule – to promote the integrity of allocation and to reduce the possibility of releasing assignments before a person has been verified as ready for that assignment [53]. Participants are counted as randomized as soon as the intervention allocation is issued and continue to be counted according to the group assigned, regardless of the course of intervention, according to intention-to-treat principles [53]. Participants are encouraged not to reveal their group allocation to the scientific field interviewers as the trial proceeds.

### Outcome indicators and measures

The BCHCP trial outcome indicators and measures were explicitly chosen to enable replication of some of the more robust US trial findings, while also addressing potential explanatory factors and indicators of salience for BC policy and practice. Tables 4 and 5 provide an overview of the measures at each assessment point. Names of validated scales/instruments are provided with citations, including those from which individual items were selected for this trial.

**Table 4:** Maternal outcome indicators and assessment points

Outcome indicators (Measures^1^)	Baseline	34 weeks	Birth event	Two months	10 months	18 months	24 months
*Demographics and socioeconomic status*							
Age, racial/cultural group, language [63]	x	x		x	x	x	x
Education + employment [63]	x	x		x	x	x	x
Income + financial supports (Family Resource Scale Revised- Basic Needs) [63, 64]	x	x		x	x	x	x
Housing/residential stability [63]	x	x		x	x	x	x
Relationship status + demographics [11]	x	x		x	x	x	x
*Maternal health and functioning*							
Obstetric history [11]	x	x	x				
History of abuse or neglect (Childhood Trauma Questionnaire; Childhood Experiences of Violence Questionnaire) [65, 66]	x						
General health + long-term illness [67, 68]	x						x
Self-efficacy (Generalized Self-Efficacy Scale; Pearlin Mastery Scale) [69, 70]	x	x		x	x	x	x
Psychological wellbeing (Mental Health Inventory) [71]	x						
Anxiety + depression (Kessler Psychological Distress Scale) [72]	x	x		x	x	x	x
Prenatal nicotine + alcohol use^2^ [73, 74]	x	x					
Prenatal drug use [73]	x	x					
Intimate-partner violence (Composite Abuse Scale) [75]	x	x		x	x	x	x
Executive functioning (Stroop Colour-Word Task; Trail Making Test) [76, 77]	x						
Cognitive ability (Shipley-II) [78]	x						
Substance misuse [11]				x	x	x	x
Antisocial behaviour [79, 80]	x	x		x	x	x	x
Contraceptive use [11]				x	x	x	x
Subsequent pregnancies^2^ [11]				x	x	x	x

^1^Name of validated measure (items partially sourced from other measures indicated by citation)

^2^Secondary outcome indicators

**Table 5:** Parenting and child outcome indicators and assessment points

Outcome indicators (Measures^1^)	Baseline	34 weeks pre	Birth event	Two months	10 months	18 months	24 months
*Parenting behaviours and beliefs*							
Breastfeeding initiation + duration [11]				x	x	x	
Provision of safe + nurturing home environment [11]				x	x	x	x
Parenting attitudes/beliefs (Adolescent-Adult Parenting Inventory II) [81, 82]						x	
Child exposure to 2nd hand smoke [83]				x	x	x	x
*Neonatal health*							
Pre-term birth			x				
Birth weight			x				
Apgar scores (1 + 5 min)			x				
Intensive care admission(s)			x				
*Child health and development*							
General health + long-term illness [67, 73]							x
Immunizations				x	x	x	x
Language (Ages and Stages) [84]					x	x	
Language + cognition^2^ (Bayley SID-III; MacArthur-Bates Communication Development Inventories) [85, 86]							x
Behaviour^2^ (Child Behavior Checklist) [87, 88]							x
Healthcare encounters for injuries^3^				x	x	x	x
Substantiated abuse or neglect				x	x	x	x
*Maternal and child service access and use*							
Prenatal programs	x	x					
Primary + secondary healthcare	x	x		x	x	x	x
Specialist care, e.g., mental health	x	x		x	x	x	x
Financial/educational assistance	x	x		x	x	x	x
Other services, e.g., housing	x	x		x	x	x	x
Parenting programs	x	x		x	x	x	x
Early child development programs				x	x	x	x
Other services	x	x		x	x	x	x
Barriers to essential services	x	x		x	x	x	x

^1^Name of validated measure (items partially sourced from other measures indicated by citation)

^2^Secondary outcome indicators

^3^Primary outcome indicator

#### Primary outcome indicator

The primary outcome indicator is child injuries from birth through 24 months. Independent data on healthcare encounters for child injuries will be obtained from personal records made available through secure data-sharing agreements with the BC Ministry of Health, which accesses and holds data on healthcare encounters across the province. These data sources include: BC Medical Services Plan physician billing records, which capture outpatient/community and emergency encounters (including dates of service and diagnoses); National Ambulatory Care Reporting System records, which capture emergency encounters (including dates of service, presenting complaints, discharge diagnoses and discharge dates); and Discharge Abstract Database records, which capture hospital encounters (including diagnoses and admission and discharge dates). These databases use diagnostic codes from the World Health Organization’s International Classification of Diseases (ICD) (9th and 10th editions). The trial will use the injury diagnosis codes and cause of injury codes pertaining to external injuries (e.g., fracture, burn, ingestion, open wound).

#### Secondary outcome indicators

The secondary outcome indicators are: prenatal nicotine and alcohol use; child cognitive development (language and cognitive ability) at age two years; child behaviour (internalizing and externalizing problems) at age two years; and the incidence of subsequent pregnancies by 24 months postpartum. (See Tables 4 and 5.)

### Frequency and nature of data collection to assess trial outcomes

Data are being collected via multiple methods and from multiple sources including: maternal self-report surveys, maternal and child observational and cognitive testing, and data linkage as well as data extracts. Scientific field interviewers verbally administer survey items and record the responses, to ensure comprehension and accuracy. For maternal self-report items prone to response bias, e.g., prenatal substance use and IPV exposure, questions are being administered using audiotaped recordings, with participants responding confidentially on paper and responses being placed in sealed envelopes. Data-sharing agreements were established between the Children’s Health Policy Centre at SFU and the BC Ministries of Health and Children and Family Development to facilitate data linkage regarding personal data (e.g., healthcare encounters for child injuries, substantiated cases of child maltreatment) documented in provincial datasets as well as NFP nurse assessment data. Data on neonatal outcomes are being provided through the BC Perinatal Database Registry.

**Fig. 1:**
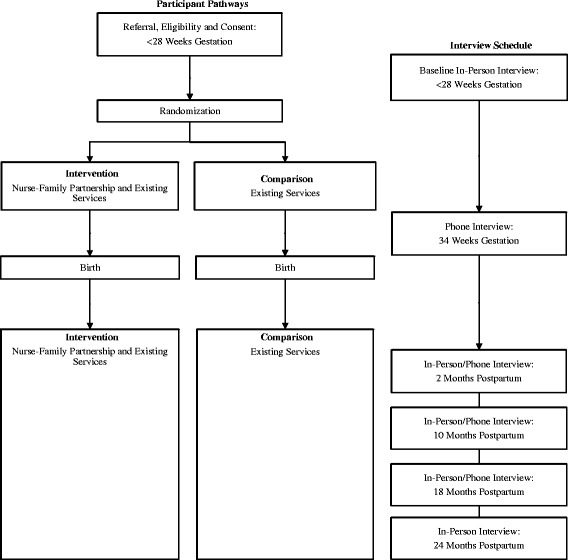
Participant pathways and interview schedule

Women participate in six data collection interviews from early in pregnancy until their child reaches two years of age. The interviews are conducted by scientific field interviewers: in person at baseline; by telephone at 34 weeks gestation; in person or by telephone at two months (to strengthen rapport and further encourage retention); in person or by telephone at 10 and 18 months postpartum; and in person at 24 months postpartum. Interviews typically take between 60 min (telephone) and 120 min (in person). Figure 1 depicts the participant pathways and interview schedule.

Scientific field interviewers have baccalaureate or master’s degrees, but no clinical training (in contrast with other recent NFP RCTs) [24, 25]. The Scientific Team therefore provides rigorous training that prepares interviewers to: rigorously assess socioeconomic eligibility criteria; manage large participant caseloads spanning wide geographic areas; track participants who have limited phone access and who move frequently; conduct in-depth interviews and record sensitive personal data in challenging community settings; follow intention-to treat protocols; build and sustain participant engagement with the trial; and discern and report adverse events.

Both prior to and during the trial, the Scientific Team at SFU provides research orientation sessions to NFP PHNs, supervisors, the BC Ministry of Health Provincial NFP Coordinator, and relevant Health Authority public health staff (e.g., managers, directors and administrative support staff) on “best-practice” procedures for RCT implementation.

### Participant retention

Based on a review of the research literature, the Scientific Team at SFU developed a detailed retention protocol [54–56]. This protocol includes the following steps and elements.

- Trial identification materials were developed including trial logo, business cards, letterhead and website;
- Participants are assigned to the same scientific field interviewer for the duration of the trial, wherever possible, to maintain strong trial rapport;
- Interviews are held at a wide range of times including on weekends to accommodate participants’ schedules;
- Participants receive gift cards after each interview to acknowledge their time and effort;
- Participants may contact the Scientific Team using a toll-free line, e.g., if they are moving or need to change appointments;
- Participants are contacted by their scientific field interviewer shortly before, and between, interviews using preferred modes of communication, e.g., texting;
- Participants receive birthday cards for their infants; and
- Participants provide contact information for third parties (e.g., friends or family members) who scientific field interviewers may contact if they have difficulty reaching the participant.

A detailed tracking database captures participants’ progress through the trial, following Consolidated Standards of Reporting Trials (CONSORT) guidelines [57]. Informed consent is also being obtained to contact participants for long-term follow up – to set the stage for evaluating NFP’s impact across early childhood and beyond, including economic evaluation. The trial was also designed to not reveal the purpose to participants, but rather, to encourage them to share their experiences as young pregnant women and new mothers living in BC.

### Statistical analysis

A statistical analysis plan was developed for this RCT, providing a detailed summary of the data analysis methodology to be used. The trial statistician and Scientific Team will verify and approve the statistical analysis plan prior to any data being examined, to prevent biasing the analyses.

#### Sample size calculation

The sample size is based on a minimal clinically meaningful difference in the reduction of the number of physician encounters for injuries per child in community/outpatient, emergency and hospital settings – the RCT’s primary outcome indicator. In the BCHCP trial population, the base rate of injuries per child from birth through two years is expected to be 0.30 (30 %), based on aggregate-level BC data from a 10-year cohort of children born to first-time mothers receiving income assistance, provided by the BC Ministry of Health for the trial (unpublished report). This base rate is consistent with the rate of emergency room visits for injuries (accidents and ingestions) during children’s second year of life in a previous NFP trial in Elmira, US [14]. By consensus, the Scientific Team set the minimal clinically-meaningful reduction in risk as a relative risk of 0.70, i.e., a reduction from 0.30 (30 %) in the comparison group to 0.21 (21 %) in the intervention (NFP) group. With the probability of a type I error rate (alpha) set at 0.05 (2-tailed), a sample size of n = 495 per group is needed to detect a 30 % relative risk reduction with type II error (beta) set at 0.20 (power = 80 %). Presuming 5 % attrition only for the primary outcome indicator, due to accessing personal data from provincial datasets to measure this indicator, the estimated total sample size is 1040.

#### Statistical analysis

The primary and secondary analyses will be conducted on an intention-to-treat basis. We will use the most recent version of MLwiN multilevel modeling software for the statistical analyses of our primary and secondary outcomes [58]. Equations for the multilevel regression models are stated in the statistical analysis plan for outcomes with Poisson and Normal distributions. Prior to analysis, data will be checked for outliers, inconsistencies and possible transformation. We predict that our sample size will be large enough for our statistical tests to be robust in the face of non-normally distributed variables. To reduce bias and loss of statistical power in our analysis of primary and secondary outcome indicators, we will use multiple imputation to estimate values for missing variables.

Descriptive statistics regarding baseline demographic, socioeconomic and personal risk variables will be used to identify any variables with a standardized difference between the groups of *d* > 0.05 for consideration as a candidate variable for control in the analyses. We do not expect differences among Local Health Areas in the implementation of NFP due to the rigorous education and manualized delivery of the program following core model elements. Nevertheless, we will use Local Health Area (site) for stratification purposes and test for between-site differences in NFP versus existing services outcomes.

## Discussion

The purpose of this trial is to determine NFP’s effectiveness compared with BC’s existing services in improving children’s mental health and early development and mother’s life circumstances. NFP holds singular promise for young, first-time mothers and their children who are experiencing socioeconomic disadvantage – a population with high needs which has often been underserved. Yet NFP’s effectiveness has not been tested previously in Canada. The BCHCP is therefore laying the foundation for NFP to be evaluated and adapted for sustained use in BC, and potentially across Canada, should RCT findings be positive.

NFP is a time-limited intervention occurring prenatally and over the first two years of life. However, evidence from the US NFP trials suggests that important benefits may either grow stronger over time, or be revealed with new developmental advantages as children grow older. For example, some of NFP’s most compelling US findings have been demonstrated five to 20 years after the intervention ended including: reductions in child anxiety, depressive and substance misuse symptoms; reductions in serious antisocial behaviour in adolescence; demonstrations of cost-effectiveness; and decreased child and maternal mortality due to preventable causes [5-20]. Consequently, this RCT is intentionally designed to permit long-term follow up – through both robust retention efforts and the inclusion of measures predictive of longer-term outcomes, e.g., child cognitive development and behaviour. Accordingly, any inference about NFP’s success or failure based on the trial outcomes when children reach age two years will not rule out the possibility that findings may be overturned in subsequent developmental periods.

Perhaps the most unique aspect of the BCHCP is that it involves close collaborations with policymakers and practitioners within BC’s public health system, including the Ministry of Health, the Ministry of Children and Family Development and the participating Health Authorities. These research-policy-practice collaborations encourage integrated and reciprocal “knowledge exchange” throughout the trial such that findings, should they be positive, will be readily applicable in policy and practice settings across BC, and Canada. The Scientific Team hopes that by generating new evidence through the BCHCP RCT, BC policymakers and practitioners – and all Canadians – will be encouraged to take new steps to improve children’s mental health and development starting very early in the lifespan, while also improving mother’s circumstances.

## Abbreviations

BC, British Columbia; BCHCP, British Columbia healthy connections project; IPV, intimate-partner violence; NFP, nurse-family partnership; PHN, public health nurse; RCT, randomized controlled trial; SFU, Simon Fraser University; US, United States
